# Vaccine Design and Vaccination Strategies against Rickettsiae

**DOI:** 10.3390/vaccines9080896

**Published:** 2021-08-12

**Authors:** Anke Osterloh

**Affiliations:** Research Center Borstel, Parkallee 22, 23845 Borstel, Germany; aosterloh@fz-borstel.de; Tel.: +49-4537-188-4822

**Keywords:** rickettsiae, orientia, immunity, vaccination

## Abstract

Rickettsioses are febrile, potentially lethal infectious diseases that are a serious health threat, especially in poor income countries. The causative agents are small obligate intracellular bacteria, rickettsiae. Rickettsial infections are emerging worldwide with increasing incidence and geographic distribution. Nonetheless, these infections are clearly underdiagnosed because methods of diagnosis are still limited and often not available. Another problem is that the bacteria respond to only a few antibiotics, so delayed or wrong antibiotic treatment often leads to a more severe outcome of the disease. In addition to that, the development of antibiotic resistance is a serious threat because alternative antibiotics are missing. For these reasons, prophylactic vaccines against rickettsiae are urgently needed. In the past years, knowledge about protective immunity against rickettsiae and immunogenic determinants has been increasing and provides a basis for vaccine development against these bacterial pathogens. This review provides an overview of experimental vaccination approaches against rickettsial infections and perspectives on vaccination strategies.

## 1. Introduction

Rickettsiae are small obligate intracellular bacteria of the family of Rickettsiacea that cause febrile potentially fatal diseases in humans. The family of Rickettsiacea consists of two genera, Rickettsia and Orientia. *Orientia* (*O.*) *tsutsugamushi* has long been considered the only member of the latter. Meanwhile, two additional species have been recently identified (candidatus *O. chuto* [1] and candidatus *O. chiloensis* [2]). The genus Rickettsia is further divided into four major groups of species according to phylogenetic relationship and way of transmission. The majority of rickettsial species belong to the spotted fever group (SFG). More than 20 species of this group have been identified so far. Prominent representatives of this group are *R. rickettsii*, the causative agent of Rocky Mountain spotted fever (RMSF), and *R. conorii* that causes Mediterranean spotted fever (MSF). The second group is the typhus group (TG) of rickettsiae that has two members, *R. prowazekii*, the causative agent of epidemic typhus, which was and is a serious threat in times of war, and *R. typhi* that causes endemic or murine typhus. The third group of pathogenic rickettsiae is the transitional group (*R. felis*, *R. akari*, *R. australis*), while the fourth group, the ancestral group (*R. bellii* and *R. canadensis*), is non-pathogenic.

Transmission of rickettsiae to vertebrates generally occurs through arthropods that carry the bacteria as endosymbionts in the gut epithelium. Once infected, the arthropod stays infected for life and transmits the bacteria transovarially and transstadially to the next generation. *R. prowazekii* is transmitted from human to human via the body louse (*Pediculus humanus*), while all other so-far-known rickettsial species are transmitted to humans via ectoparasites from rodents, predominantly from rats and mice. Rodents serve as a natural reservoir and play a key role in the distribution of rickettsial infections. It is, therefore, not surprising that rickettsial infections generally occur worldwide, which is especially true for TG rickettsiae while other rickettsial species appear endemic in certain areas of the world (Table 1). All SFG rickettsiae are transmitted by ticks (genera Dermacentor, Rhipicephalus, Amblyomma, Hyalomma, or Ixodes), while mites are the vectors for the transmission of orientia species (*Leptotrombidium deliense*) and *R. akari* (house mouse mite Liponyssoides sanguineus). The only so-far-known rickettsial species that are transmitted by fleas are *R. typhi* (predominantly the rat flea *Xenopsylla cheopis*) and *R. felis*, the causative agent of cat-flea typhus (the cat flea *Ctenocephalides felis*) [3].

In the case of TG rickettsiae, transmission occurs via deposit of feces from an arthropod that has been infected by ingesting blood from an infected rodent. The bacteria are then scratched into the wound. SFG rickettsiae can also be transmitted via the bite of an infected tick. After entering the vertebrate skin, rickettsiae first infect phagocytic cells and then spread into endothelial cells (ECs) that form the inner wall of the blood vessels and represent the dominant target cells [4,5]. Rickettsiae replicate free in the cytosol of infected ECs and are released by different mechanisms. SFG rickettsiae can induce focal lysis of the cellular membrane to be released and spread to adjacent cells [6,7]. TG rickettsiae multiply until lysis or burst of the cell [7], and orientia performs a kind of budding similar to viruses [8]. Free bacteria then infect adjacent ECs leading to local blood leakages and inflammatory reactions that can become visible as an eschar at the site of entry in the infection with some SFG rickettsiae (e.g., *R. conorii* but not *R. rickettsii*), orientia, and *R. prowazekii* but not *R. typhi*. The bacteria further systemically spread throughout the body via the bloodstream and can enter nearly all organs, where they also infect other cells, predominantly monocytes/macrophages (MΦ) [4,5,9]. These cells are considered to function as a niche for bacterial replication and to play a central role as a vehicle for dissemination [10,11,12]. Disseminated rickettsial infections cause a characteristic skin rash in approximately 60% of the patients, which is due to local blood leakages and inflammatory reactions.

The disease that is caused by the infection with different rickettsial species appears quite similar but with different severity. Patients usually develop high fever and suffer from headaches and abdominal pain. Because of the systemic distribution of the bacteria in the body, the infections can lead to multiple organ pathology, including pneumonia, meningoencephalitis, nephritis, myocarditis, hepatic damage, and other complications that can be fatal. The highest mortality is observed for the infection with *R. rickettsii* (>20% without antibiotic treatment and nowadays 1–7% [13]) followed by epidemic typhus caused by *R. prowazekii* that usually appears under poor hygienic conditions and lack of medical care (15–30%) [14,15]. Due to the relatively high mortality, *R. rickettsii* and *R. prowazekii* are classified as potential biological weapons.

Disease surveillance data are rare because diagnosis is still problematic. In a recent study from the Chinese Center for Disease Control, it was described that the incidence of orientia infections increased from 0.09/100,000 in 2006 to 1.6/100,000 in 2016 and that the disease that was originally endemic to southern China expanded to all provinces [16]. In the U.S., an increase in spotted fever rickettsioses is observed, and also endemic typhus caused by *R. typhi* appears with steadily increasing incidence predominantly in Southern California, Texas, and Hawaii [17,18,19]. The Centers for Disease Control (CDC) recorded 738 cases of endemic typhus in Texas in 2018 compared to 222 in 2013 and 157 in 2008 (https://www.dshs.texas.gov/IDCU/disease/murine_typhus/Typhus-2008-2018.pdf, accessed 6 August 2021).

Generally, infections with *R. typhi* are highly endemic in coastal tropical and subtropical regions in low-income countries in Asia [20,21,22,23], Africa [24], and South America, e.g., Mexico [25]. In Laos, it was found that the infection with *O. tsutsugamushi* and *R. typhi* was responsible for a high proportion of central nervous system infections (12% and 11%, respectively) with a high mortality rate [26]. In Europe, rickettsial infections are predominantly detected in travelers who acquired the infection abroad. Nonetheless, e.g., *R. typhi* also appears in Europe (Greece, Cypres, Spain, Portugal [27,28,29,30,31,32,33,34,35]). Especially homeless people are at enhanced risk. In France, the seropositivity of the homeless in Marseille dramatically increased in the past 20 years from 0.054% in the years 2000–2003 to 22% in the years 2010–2013 [36]. In addition to that, sporadic outbreaks of rickettsial infections occur that are usually associated with poor hygienic conditions. After the earthquake in Nepal in 2015, increasing cases of febrile illnesses were observed in refugee camps. These were revealed to be caused by the infection with *O. tsutsugamushi* and had relatively high mortality of 5.7% [37]. Since then, the transmission of *O. tsutsugamushi* is ongoing in Nepal. Other examples are the large outbreak of epidemic typhus during civil war in Burundi in 1995 in a jail and in refugee camps [38,39,40] and a smaller outbreak of epidemic typhus in Russia in 1997 [41].

A major problem in the recognition and treatment of rickettsial diseases is that diagnostic methods are still limited, not standardized, expensive, and often not accessible. Misdiagnosis, delayed antibiotic treatment, and treatment with inappropriate antibiotics often result in more severe or fatal disease. This was also the reason for the high mortality of *O. tsutsugamushi* infections after the earthquake in Nepal, where clinicians treated the patients with cefexime or ceftraiaxone and imipenem [37]. Rickettsiae and orientiae respond to only a few antibiotics (doxycycline, rifampin, chloramphenicol), with doxycycline being the treatment of choice. The development of antibiotic resistance is a great threat, and also, in cases of doxycycline intolerance, effective alternative antibiotics are missing. Finally, some rickettsial species can persist and reoccur despite antibiotic treatment, which is well known for *R. prowazekii*. These bacteria can reappear several years after primary infection and cause Brill-Zinsser disease [42]. The persistence of other rickettsial species in humans (*R. rickettsii* [43,44], *R. typhi* [12], as well as of *O. tsutsugamushi* [45]) is anticipated but has not yet been proven. At least for *O. tsutsugamushi*, relapse of patients that had been initially treated with antibiotics and recovered from the disease was observed months to years after the infection [45].

For the reasons mentioned above, a prophylactic vaccine against rickettsial infections is urgently needed. Vaccine design requires the understanding of protective immunity and also immunopathological reactions. This review provides a brief overview of current knowledge on immunity and immunopathology in rickettsial infections and focuses on experimental vaccination approaches and perspectives on vaccine design against these bacteria.

## 2. Adaptive Immunity Is Essential for Defense against Rickettsial Infections

Immunodeficient mice that lack T and B cells are highly susceptible to the infection with various rickettsiae [12,46,47], while common laboratory wild-type mouse strains such as C57BL/6 and BALB/c mice are resistant. This clearly indicates that adaptive immunity is essential for defense against rickettsiae.

Cytotoxic CD8^+^ T cells generally play a major role in defense against most intracellular pathogens and are also activated in the infection of mice with various rickettsiae as well as *O. tsutsugamushi*. The cells express IFNγ and show enhanced cytotoxic activity [48,49,50,51,52]. In case of the infection with SFG (*R. rickettsii*, *R. conorii*), transitional rickettsiae (*R. australis*), and *O. tsutsugamushi*, CD8^+^ T cells seem to be indispensable for defense as reflected by the observations that CD8^+^ T cell-deficient or depleted mice show reduced survival, enhanced bacterial burden and pathology in the infection with *R. conorii, R. australis* and *O. tsutsugamushi* [48,51,52,53]. Furthermore, mice are protected against the infection with *R. conorii* as well as with *O. tsutsugamushi* after the adoptive transfer of immune CD8^+^ T cells [51,53]. In the case of *O. tsutsugamushi*, a long-lasting CD8^+^ T cell response seems to be important for the control of persisting bacteria as the depletion of CD8^+^ T cells months after the infection leads to reactivation of the bacteria in mice [51]. Similar seems to be true for the infection with *R. typhi* that has also been demonstrated to persist in C57BL/6 mice as well as in BALB/c mice [12]. In concordance with the persistence of *R. typhi*, both mouse strains show a long-lasting CD8^+^ as well as a CD4^+^ T cell response that is even sporadically reactivated in BALB/c mice over time [49,50], indicating that both cell populations are needed for protection against recurrence.

The protective activity of CD8^+^ T cells in defense against the infection with SFG and transitional rickettsiae as well as *O. tsutsugamushi* mainly relies on the cytotoxic activity of CD8^+^ T cells rather than the production of IFNγ. Perforin-knockout mice that lack the cytotoxic activity of CD8^+^ T cells show higher susceptibility to *R. australis* than mice that lack the expression of IFNγ [48]. These mice also succumb to the infection with *O. tsutsugamushi* with enhanced bacterial burden in several organs [51].

The importance of CD8^+^ T cells and cytotoxic activity of these cells, however, may vary in defense against different rickettsial species. CD8^+^ T cells from *R. typhi*-infected mice also express IFNγ and granzyme B [49,50], indicating enhanced cytotoxic activity. Although it has been described that the depletion of CD8^+^ T cells leads to enhanced bacterial burden and pathology in *R. typhi*-infected C3H/HeN mice [54], other studies show that CD4^+^ T cells are sufficient for protection against this pathogen. In contrast to the infection with *R. australis*, CD8^+^ T cell-deficient C57BL/6 mice are not susceptible to the infection with *R. typhi* [50]. Moreover, the adoptive transfer of either CD8^+^ or CD4^+^ T cells into immunodeficient mice of different genetic backgrounds (C57BL/6, BALB/c) protects the animals from *R. typhi*-mediated disease and leads to bacterial elimination [49,50]. In contrast to the infection with SFG rickettsiae or *R. australis*, however, the cytotoxic activity seems to be dispensable for the protective effect of CD8^+^ T cells against *R. typhi*. This is demonstrated by the observation that BALB/c perforin-knockout mice are not susceptible to infection with this pathogen. Moreover, the transfer of Perforin-knockout CD8^+^ T cells still protects immunodeficient mice BALB/c CB17 SCID mice against *R. typhi*. Interestingly, CD8^+^ IFNγ^-/-^ T cells were revealed to be less efficient than CD8^+^ Perforin^-/-^ T cells to keep persisting *R. typhi* below the qPCR detection limit in this infection model [49,50], suggesting that the production of IFNγ by CD8^+^ T cells is more important than the cytotoxic activity for long-term control of the bacteria.

The protective capacity of CD4^+^ T cells has been demonstrated in murine infection models of SFG as well as of TG rickettsiae. Although the depletion of CD4^+^ T cells does not alter the course of the disease in the infection of C3H/HeN mice with a sublethal dose of *R. conorii*, adoptive transfer of immune CD4^+^ T cells protects these animals against challenge with a normally lethal dose of this pathogen [53]. Similarly, adoptive transfer of CD4^+^ T cells protects *R. typhi*-infected immunodeficient BALB/c CB17 SCID mice as well C57BL/6 RAG1^-/-^ mice [49,50]. In these experiments, adoptive transfer of either CD8^+^ or CD4^+^ T cells was comparably protective against *R. typhi,* although CD8^+^ T cells were clearly quicker in bacterial elimination. These findings indicate that CD4^+^ T cells are sufficient for protection against TG rickettsiae, at least *R. typhi*.

CD4^+^ T cells differentiate into T_H_1 cells that produce IFNγ and TNFα in the infection with rickettsiae and orientiae. Both cytokines activate the expression of inducible nitric oxide synthase (iNOS) in infected target cells such as ECs and MΦ, which leads to the production of bactericidal nitric oxide (NO) and bacterial killing [49,55,56]. Both cytokines are important in defense against SFG as well as TG rickettsiae and orientiae [49,57], whereby IFNγ may play a more critical role in the infection with SFG and transitional rickettsiae. For example, IFNγ-deficient C57BL/6 mice show enhanced susceptibility to *R. conorii* as well as *R. australis* [48,57]. In contrast, IFNγ-knockout BALB/c mice were shown to be resistant to *R. typhi* [49]. In this case, it can be assumed that CD8^+^ T cells and the cytotoxic activity of these cells may compensate for the absence of IFNγ. However, adoptive transfer of CD4^+^ T cells from BALB/c IFNγ-knockout mice still protects congenic immunodeficient mice against *R. typhi* [49]. These cells show a T_H_17 phenotype and produce IL-17, TNFα, and IL-22, indicating that also T_H_17 cells can confer protection against *R. typhi*. However, T_H_17 cells were also shown to have pathological effects via the coproduction of IL-17 and TNFα. Neutralization of one or the other cytokine led to enhanced survival of CD4^+^ T_H_17 recipients [49].

The role of the humoral response is considered to be less important than the cellular arm of adaptive immunity in primary defense because B cells start to produce high-affinity antibodies relatively late in the infection with rickettsiae (>day 15 in the infection with *R. typhi*, >day 16 and >day 25 in the infection with *R. conorii* and *R. africae*) [58,59]. Nonetheless, antibodies can contribute to the protection, as demonstrated by passive immunization experiments. Administration of polyclonal immune serum from *R. conorii*-infected C3H/HeN mice into C3H SCID mice protects the animals against a lethal challenge with *R. conorii* [47]. Even in already infected C3H SCID mice, the application of immune serum leads to prolonged survival and reduced bacterial load [47]. Targets of the humoral response are likely surface proteins of the bacteria that are easily accessible for antibodies. Bound to surface proteins, antibodies can opsonize the bacteria for the uptake by phagocytes, inhibit the receptor-mediated uptake of the bacteria into target cells, or induce complement activation and bacterial destruction.

## 3. Immunopathology in Rickettsial Infections

Only a few descriptions of immunopathological mechanisms in rickettsial infections are found in the literature. These relate to the infection with *O. tsutsugamushi* and *R. typhi*.

*O. tsutsugamushi* enters MΦ and replicates in these cells. Unlike many other intracellular bacteria, it induces an M1 phenotype. *O. tsutsugamushi*-infected human as well as murine MΦ produce NO as well as enhanced levels of inflammatory cytokines including IL-1β and TNFα [60,61,62]. It has recently further been shown that *O. tsutsugamushi* not only survives and replicates in murine MΦ despite the presence of NO, but that NO even enhances bacterial replication in MΦ [63]. This M1 polarization likely depends on TLR2 as *O. tsutsugamushi* has been demonstrated to use this receptor to induce the secretion of TNFα and IL-6 in DCs [64]. Although IL-1β, TNFα, and other pro-inflammatory cytokines that are produced by *O. tsutsugamushi*-infected MΦ and DCs contribute to a protective T_H_1-polarized immune response, they also induce inflammatory reactions in the tissue environment. Therefore, M1 MΦ are considered to be largely responsible for tissue pathology that is observed in scrub typhus patients [62]. In support of this, it was shown that *O. tsutsugamushi*-infected Toll-like receptor 2 (TLR2)^-/-^ C57BL/6 mice were even better protected from lethal infection compared to wild-type mice and showed lower bacterial burden and milder symptoms of disease [64]. These observations indicate that the inflammatory effects of MΦ and maybe also DCs are responsible for more severe disease and that TLR2 is dispensable for the induction of protective immunity.

In addition to that, also CD8^+^ T cells have been shown to be involved in MΦ-mediated tissue pathology in experimentally infected mice. *O. tsutsugamushi*-infected C57BL/6 mice show lung inflammation and hepatic injury. The latter was shown to be dependent on the infiltration of CD8^+^ T cells, followed by MΦ infiltration. Furthermore, inflammation of the lung could be attributed to CD8^+^ T cells [51]. These observations indicate a positive feedback mechanism between activated CD8^+^ T cells and MΦ, most likely via CD8^+^ T cell-derived IFNγ as an activator of MΦ, that accelerates the inflammatory response and leads to enhanced pathology in scrub typhus disease. On the other hand, *O. tsutsugamushi*-infected CD8^+^ T cell-deficient mice show enhanced lethality and uncontrolled bacterial growth, although these mice produce enhanced IFNγ levels and show stronger MΦ responses in the organs, which is correlated to enhanced tissue damage [51]. In this case, it is speculated that the absence of CD8^+^ T cells results in enhanced activation of CD4^+^ T cells as a compensatory mechanism and that IFNγ that is produced by these cells drives MΦ activation and pathology. Together these observations indicate that especially IFNγ, produced by either CD8^+^ or CD4^+^ T cells, can enhance tissue pathology by activating MΦ.

Other, yet unclear mechanisms, that may contribute to pathology are the development of anti-nuclear antibodies (ANAs) that are observed in around 40% of scrub typhus patients [65] and the release of IL-17. Levels of IL-17 are generally enhanced in scrub typhus patients and higher in patients who suffer from headaches [66], suggesting a causal relationship.

Inflammatory MΦ can clearly enhance tissue damage. Other cells of the innate immune system that can be involved in pathology also include neutrophils. *R. typhi*-infected immunodeficient BALB/c CB17 SCID mice develop severe liver necrosis. In the absence of neutrophils upon depletion of this cell population, the mice succumb to the infection with the same kinetics and develop comparable bacterial loads in all organs as control groups, but the depletion of neutrophils completely prevents liver damage [46]. In addition to neutrophils, also MΦ play clearly a role in pathology in the infection with *R. typhi*. In contrast to the infection with *O. tsutsugamushi*, however, MΦ hardly respond to *R. typhi* in vitro and do not show an M1 phenotype per se. They do not produce inflammatory cytokines or NO upon infection with *R. typhi* but exclusively upregulate major histocompatibility class I (MHCI) [46]. This indicates that the bacteria are either not recognized in a classical manner, e.g., via TLR, or that the bacteria actively suppress MΦ activation. In BALB/c CB17 SCID mice, *R. typhi* predominantly resides in MΦ [46]. MΦ also expressed iNOS and produced, together with NK cells, high amounts of IFNγ. The expression of iNOS, however, was restricted to those MΦ that did not harbor *R. typhi* [46], indicating that activation of these cells appears through indirect mechanisms, probably endogenous danger signals that are released from damaged tissue or IFNγ produced by NK cells. In another model of *R. typhi* infection (immunodeficient C57BL/6 RAG1^-/-^ mice), the bacteria are also found predominantly in MΦ. These mice develop severe central nervous system (CNS) inflammation, which is due to massive accumulation and activation of microglia as well as to the presence of infiltrating MΦ. In contrast to BALB/c CB17 SCID mice, these infiltrating MΦ carry *R. typhi* and express iNOS [12]. Here, the expression of iNOS and CNS inflammation is largely enhanced by the adoptive transfer of immune CD4^+^ T cells but not CD8^+^ T cells relatively late in the infection, although the bacteria were efficiently eliminated by both cell populations [50]. This observation suggests that brain inflammation in this model is mainly due to immunopathology rather than cellular destruction by the bacteria themselves. This MΦ activation can be put down to the release of IFNγ by CD4^+^ T cells [50] and again, as in the infection with *O. tsutsugamushi*, demonstrates that the T cell-derived IFNγ-MΦ axis, although essential for protection, has pathological side effects.

Whether immunopathology plays a role in the infection with SFG rickettsiae is unclear. *R. conorii* has been demonstrated to induce an MΦ M2 phenotype with reduced production of reactive oxygen species (ROS), among other effects that inhibit pro-inflammatory signaling and M1 polarization [10]. This argues against a major contribution of MΦ to pathology in the infection with SFG rickettsiae agents.

## 4. Vaccination against Rickettsiae with Whole-Cell Antigen (WCA)

The first attempts of immunization against rickettsiae were made with inactivated intact bacteria that were either produced in arthropods, embryonated chicken eggs, embryonal chicken fibroblasts, or infected animals. The first whole-cell antigen (WCA) vaccines against *R. prowazekii* and *R. rickettsii* were produced already in the 1920s. R. L. Weigl produced *R. prowazekii* by intrarectal injection of the bacteria into lice and fed the arthropods on humans. The bacteria were prepared from the gut of the lice and inactivated in phenol. This vaccine not only protected guinea pigs from disease [67] but was also used for the vaccination of German soldiers during World War II [68]. A similar vaccine was produced by the U.S. military at the same time where *R. prowazekii* was grown in chicken egg yolk sacs and inactivated in formalin. Vaccination of U.S. soldiers during World War II ameliorated disease [69]. Another vaccine against epidemic typhus was produced by isolation of *R. prowazekii* from the lungs of infected rabbits (Castaneda vaccine) [69] or the tunica vaginalis and the peritoneum from infected rats (*Zinsser-Castaneda* vaccine) and inactivation of the bacteria in formalin [70]. The application of three doses of the Zinsser-Castaneda vaccine was sufficient to protect guinea pigs from the disease [71].

Similarly, in 1924 the first WCA vaccine against RMSF was developed by growing *R. rickettsii* in ticks that were fed on guinea pigs. The bacteria were isolated by triturating the arthropods and inactivated in phenol and formalin [72]. In another attempt, *R. rickettsii* was grown and isolated from embryonated chicken eggs. In this way, the *Cox* vaccine, also inactivated *R. rickettsii*, was produced [73]. Administration of these inactivated bacteria leads to milder disease in humans [73] and the production of antibodies but does not completely prevent infection and disease [74]. Similarly, formalin-inactivated *R. rickettsii* that were produced by the U.S. military in the 1970s in embryonal chicken fibroblasts [75,76] protected cynomolgus and rhesus monkeys after two times immunization [77,78]. This vaccine ameliorated disease in humans but did not prevent the infection [79].

Formalin-inactivated bacteria were also used for first attempts for vaccination against scrub typhus. Here, *O. tsutsugamushi* was isolated from homogenized formalin-fixed lungs from infected cotton rats [80,81] or purified, followed by formalin-inactivation [82]. The vaccination with such inactivated *O. tsutsugamushi* only partially protected mice against challenge with the homologous bacterial strain in early studies from the 1940s [83,84], while a more recent study described protection of C3H/HeN mice against challenge with the homologous strain and long-term immunity (>8 months) [85]. Vaccination of humans, however, did not prevent infection and disease [82].

Phenol or formalin treatment of the bacteria can result in the modification of antigenic determinants, which could explain the ineffectiveness of such vaccines. Other possibilities of inactivation that can preserve antigenic structures include heat-inactivation at 56 °C or irradiation. Irradiated *O. tsutsugamushi* was found to protect mice against challenge with homologous bacteria [86,87,88], and heat-inactivated *R. rickettsii* protected dogs from severe RMSF [89], indicating a higher protective capacity compared to formalin-fixed bacteria.

Another possibility is the use of avirulent or attenuated bacteria. Examples are the vaccination with a low-virulence strain of *O. tsutsugamushi* that efficiently induces immunity in humans [90] and vaccination with *R. prowazekii* strain Madrid E. This strain was isolated during World War II and lost virulence during several passages through embryonated chicken eggs. *R. prowazekii* Madrid E has been successfully used for the vaccination of humans and induces long-term immunity up to approximately five years [91,92]. The use of such strains, however, bears the risk of reversion to a pathogenic form. Avirulence of *R. prowazekii* Madrid E is due to a mutation in the methyltransferase that is responsible for methylation of surface proteins, including OmpB. In *R. prowazekii* Madrid E, this protein, as well as other surface proteins, is hypomethylated [93]. After passage through mice, *R. prowazekii* Madrid E shows a reversion of this mutation, and reisolates of these bacteria (*R. prowazekii* Evir) are pathogenic again [94].

Stably attenuated rickettsial strains that can be produced by the introduction of mutations or the deletion of virulence genes may provide a safer way of vaccination. Genetic manipulation of rickettsiae is possible, and an attenuated strain of *R. prowazekii* was produced by site-directed knockout of the gene encoding for phospholipase D [95] that is involved in phagosomal escape [96]. Guinea pigs that were immunized with these bacteria were protected against lethal challenge with virulent *R. prowazekii* [95].

Although attenuated mutant or knockout strains are promising vaccine candidates, virulence factors that are essential for infectivity and pathogenicity are largely unknown and still need to be identified. These may include other proteins that are involved in bacterial adherence and invasion, e.g., OmpA and OmpB. Knockout of OmpA, however, did not influence the infectivity of *R. rickettsii* in guinea pigs [97]. Another problem with this kind of vaccine is that large-scale production is time-consuming and expensive, and hardly applicable for the immunization of a larger portion of people in affected areas.

Therefore, other strategies and vaccines that can be produced much more easily at larger amounts are needed.

## 5. Immunogenic Determinants and Vaccine Candidates

The development of such vaccines requires the knowledge of immunodominant rickettsial antigens that can induce protective adaptive immune responses as well as the elucidation of the optimal way of antigen delivery.

So far, only a few rickettsial antigens have been described. Most of these have been identified because they are recognized by antibodies. The most prominent ones belong to the surface cell antigen (Sca) autotransporter family (Sca 0–5) that are involved in bacterial adherence and uptake into target cells. Among this family, especially the outer membrane protein A (OmpA/Sca0), which is not expressed by TG rickettsiae, and OmpB/Sca5 represent immunodominant surface antigens that are recognized by antibodies and also by T cells in infected mice and patients [98]. Passive immunization with antibodies against OmpA and OmpB protects C3H/HeN mice and guinea pigs from normally lethal challenges with *R. rickettsii* and *R. conorii* [99,100,101,102] and even C3H SCID mice against infection with *R. conorii* [47]. Antibodies against OmpA and OmpB have been shown to enhance the uptake of *R. conorii* by phagocytic cells [103], to inhibit adherence of *R. rickettsii* to L929 cells [104] as well as to mediate complement-mediated killing of the bacteria [102] so that all three mechanisms may contribute to protection.

The majority of other antigens described in the literature are also surface-expressed proteins that are predominantly recognized by antibodies. Exceptions are Sca4 and the molecular chaperone GroEL, both of which are cytosolic proteins. GroEL, however, has also been demonstrated to be surface-exposed in SFG as well as TG rickettsiae and to be recognized by antibodies [105,106] that can enhance bacterial uptake into phagocytes [106]. Table 2 provides an overview of all so-far-identified rickettsial antigens.

Only a few of these proteins (OmpA, OmpB, Adr2, YbgF, and ScaA from orientia) have been shown to be also detected by T cells. Experimental evidence for the recognition by B and T cells of these antigens is reviewed elsewhere in more detail [113]. Generally, data on antigen-specific T cell responses, however, are rare, and immunodominant antigens that are recognized by CD4^+^ and/or CD8^+^ T cells still need to be identified. Experimentally, this can be achieved by immunoprecipitation of MHCII from professional APCs such as DCs and MΦ treated with live or inactivated bacteria, or of MHCI from cells infected with rickettsiae. Bound peptides can then be identified by mass spectrometry. Such studies, however, are still missing.

Other possibilities include the use of bioinformatic tools. Meanwhile, several bioinformatic tools are available that can assist in the determination of antigenic proteins and vaccine design by predicting the general immunogenicity of a protein (Vaxign and Vaxitope [114], VaxiJen [115]), potential B cell epitopes (ANTIGENpro, APBpred, Epitome [116,117,118]), potential CD4^+^ and CD8^+^ T cell epitopes and the probability of MHCI or MHCII presentation (PREDBALB/c, PRED(TAP), MHCPred, NetMHCpan, NetMHCIIpan, IEBD Analysis Resource, RANKPEP and SYFPEITHI [119,120,121,122,123,124,125,126]). In addition, knowledge of the predicted localization of a protein (SOSUIGramN, pSORTb, SignalP, SecretomeP [127,128,129,130]) and its function can be helpful to estimate whether it might be accessible for protective antibodies or the MHCI and MHCII presentation pathways to be recognized by CD4^+^ or CD8^+^ T cells. Bioinformatic approaches have been successfully used for the identification of five antigens from *R. prowazekii* that are recognized by CD8^+^ T cells (RP403, RP598, RP739, RP778, RP884) [131,132]. These antigens were expressed in SVEC 4–10 cells, and immunization of mice with these cells induced antigen-specific CD8^+^ T cells that produced IFNγ and granzyme B and protected the mice from lethal challenge with *R. typhi* [131,132].

Except for RP884, which is a cytosolic protein, the other four proteins are surface-exposed. Generally, it can be assumed that surface-exposed proteins or proteins that are released by the bacteria are accessible for the proteasome in the cytosol for degradation and transport into the MHCI presentation pathway for recognition by CD8^+^ T cells.

## 6. Experimental Approaches of Vaccination against Rickettsiae

**Figure 1:**
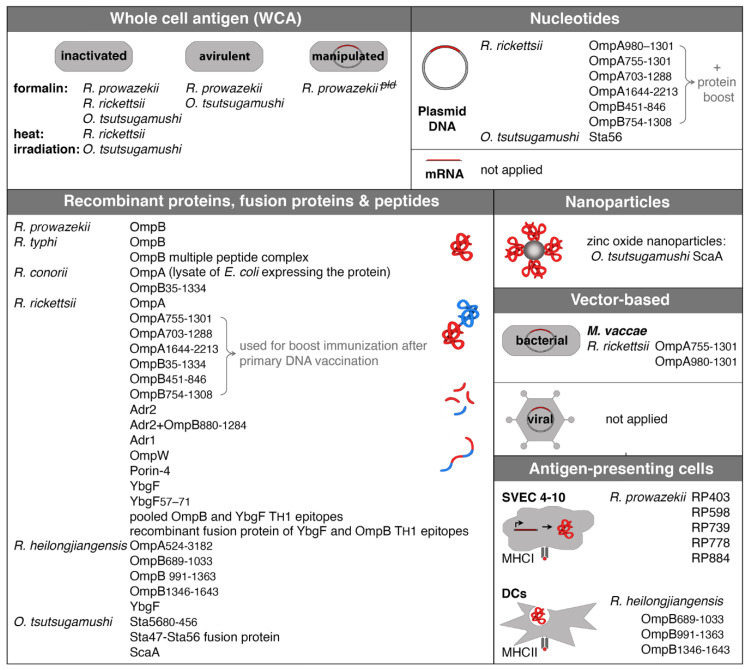
Experimental approaches of vaccination against rickettsiae. The figure shows all so far described approaches of vaccination against rickettsiae and names strains and antigens. WCA immunization was performed with either inactivated bacteria or avirulent strains. In addition, an attenuated knockout strain of *R. prowazekii* that lacks phospholipase D was generated and used for immunization in experimental infection of mice. The vast majority of vaccinations were performed with recombinant proteins, fusion proteins, or peptides. Other methods include recombinant protein coupled to nanoparticles, bacteria (*M. vaccae*), or transfected cells that express rickettsial antigens and DCs that were pulsed with recombinant protein. mRNA vaccination and vaccination with adenoviral vectors as they are used now for the immunization against SARS-Cov2 with great success have not been applied yet, but represent great new tools that should be taken under consideration.

Because of the important role of CD8^+^ T and CD4^+^ T cells in protection against rickettsiae, it stands to reason that a vaccine should address cellular immune responses, ideally in addition to the production of antibodies. While the induction of CD4^+^ T cell responses can be easily achieved by the application of recombinant protein, the difficulty in addressing CD8^+^ T cells with a vaccine lies in the delivery of the antigen into the cytosol of host cells to gain access to the MHCI presentation pathway. Antigen delivery into the cytoplasm of host cells can be achieved by different methods such as immunization with nucleotides, vector-based vaccines, or the use of APCs that express rickettsial antigens. Experimental approaches to vaccination against rickettsiae are described in the following. Figure 1 provides an overview of all experimental vaccination approaches described so far in the literature.

### 6.1. Immunization with Recombinant Proteins and Peptides

A conventional way of immunization is the application of recombinant proteins, and most approaches of vaccination against rickettsial infections in experimental animal models used either proteins, protein fragments, fusion proteins, or peptides.

OmpA and OmpB are clearly immunodominant antigens that have been extensively used for the experimental vaccination of mice. Both proteins are recognized by B as well as by T cells in the infection of animals and humans. T cells from *R. rickettsii*, *R. typhi* and *R. felis* react to MΦ that express fragments of *R. rickettsii* OmpB with the release of IL-2 and IFNγ, indicating the recognition of peptides presented by MHCI by CD8^+^ T cells and cross-reaction of T cells to conserved OmpB epitopes between SFG and TG rickettsiae [133]. This is an important point with regard to vaccination because conserved proteins such as OmpB have the potential to mediate immunity against various rickettsial species. In the case of OmpA and OmpB, recombinant proteins or protein fragments have been used for experimental immunization of animals, mainly mice or guinea pigs. Vaccination of guinea pigs with recombinant OmpA from *R. rickettsii* or truncated OmpA from *R. heilongjiangensis* protects the animals against challenges with the homologous bacteria [134,135]. In the case of the immunization with *R. heilongjiangensis* OmpA, also cross-protection against *R. rickettsii* was achieved [135]. Here, antibody production may well play a role in protection. The transfer of monoclonal antibodies against OmpA has been shown to protect immunodeficient mice from fatal infection with *R. conorii* [47]. The same was true for the application of monoclonal antibodies against OmpB [47]. In another study, it was shown that guinea pigs were protected against challenge with *R. conorii* and partially protected against the infection *R. rickettsii* upon immunization with a lysate from *E. coli* that expressed OmpA [136].

Similarly, vaccination of guinea pigs with OmpB from *R. typhi* protected the animals against challenge with this pathogen [137]. The immunization of rabbits with OmpB from *R. prowazekii* induces antibody production, and B cell epitopes were identified by the analysis of antibody binding to overlapping synthetic peptides (Table 2) [138]. An additional B cell epitope was determined from *R. typhi* OmpB and from *R. typhi* Sca1, Sca2, Sca3, and Sca4 [139]. All of these peptides are recognized by antibodies upon immunization of rabbits with a multiple peptide antigen conjugate [139].

Further, also epitopes that are recognized by CD4^+^ T cells and CD8^+^ T cells have been identified from *R. rickettsii* and *R. conorii* OmpB and from another protein, YbgF, from *R. rickettsii* (Table 2). Immunization of C3H/HeN mice with pooled CD4^+^ T cell epitopes from OmpB and YbgF or a fusion protein of these epitopes resulted in the induction of CD4^+^ T_H_1 cells that produced TNFα and IFNγ as well as in enhanced IgG1 and IgG2a production and reduced bacterial load upon infection with *R. rickettsii* [140].

The immunization of C3H/HeN mice with recombinant YbgF protein leads to enhanced proliferation and IFNγ release by both CD4^+^ and CD8^+^ T cells, prolonged IgG2a and IgG1 production, and reduced bacterial burden in the infection with *R. rickettsii* [141]. Vaccination with another recombinant immunogen from *R. rickettsii*, TolC, was less efficient than immunization with YbgF [141]. Similarly, the immunization of C3H/HeN mice with recombinant YbgF from *R. heilongjiangensis* results in reduced bacterial load upon infection with homologous bacteria [142]. The authors further demonstrate that YbgF is recognized by CD4^+^ T cells as well as by B cells in the infection with *R. heilongjiangensis* [142]. Other immunogenic proteins that have been used for experimental vaccination are Adr1, Adr2, OmpW, and Porin-4 from *R. rickettsii*. The immunization of C3H/HeN mice with recombinant Adr1, TolC, OmpW, or Porin-4 results in reduced bacterial load upon challenge with *R. rickettsii* [111]. Similarly, the immunization with recombinant Adr2 protected the animals from *R. rickettsii* infection and led to enhanced production of IFNγ by CD4^+^ T cells and TNFα by CD8^+^ T cells and increased IgG2a and IgG1 production [143]. Adr2 and OmpB have also been used in combination for experimental vaccination with the same effect [144].

In the case of orientia that phylogenetically differs from rickettsiae, three proteins have been used for experimental vaccination: Sta47, Sta56, and ScaA. *O. tsutsugamushi*-infected mice, as well as humans, develop Sta56-specific antibodies and CD4^+^ T cells [145,146,147] and antibodies against Sta47 [148]. vSta56-immunized mice produced Sta56-specific antibodies and showed enhanced proliferation of lymphocytes, which was associated with increased IFNγ and IL-2 production. Moreover, the mice were protected against challenge with the homologous *O. tsutsugamushi* strain, which produced enhanced antibody levels and lymphocytes showed increased proliferation and IFNγ and IL-2 release [149,150,151]. In a more recent study, conserved blocks of the Sta56 protein were used for the immunization of mice. This vaccination not only conferred protection against the infection with homologous bacteria but also heterologous orientia genotypes [152]. The authors further synthesized overlapping peptides from the Sta56 protein and could identify 39 peptides that are recognized by CD8^+^ T cells. Immunization with a mixture of these peptides also provided protection against lethal challenge with *O. tsutsugamushi* [152], underlining the protective activity of cytotoxic CD8^+^ T cells. In addition, a fusion protein of Sta56 and Sta47 has been used for experimental vaccination, which was, however, only partially protective against the infection with homologous orientia [153], and the vaccination of primates (*Macaca fascicularis*) with a recombinant Sta56 fragment (Sta56_80–456_) was only weakly protective and did not prevent disease and rickettsemia [154].

Other immunogenic proteins from orientia are the surface proteins Sta22, ScaA, ScaC, ScaD, and ScaE. All of these antigens have been shown to be recognized by antibodies in the infection with *O. tsutsugamushi* with a stronger response to ScaA and ScaC compared to ScaE [155,156]. In the case of Sta22, it has been shown that *O. tsutsugamushi*-infected mice develop Sta22-specific CD4^+^ T cells [155]. Of these proteins, only recombinant ScaA and ScaC have been used for experimental vaccination. Of these, only the immunization with ScaA protected mice from challenge with homologous as well as heterologous orientia strains [157]. The authors further showed that ScaA-specific antibodies inhibit the uptake of orientia by non-phagocytic HeLa cells.

### 6.2. Immunization with Antigen-Coupled Nanoparticles

There is one description in the literature where antigen-coupled nanoparticles were used for the immunization of mice against *O. tsutsugamushi*. Recombinant ScaA protein from *O. tsutsugamushi* was coupled to zinc oxide nanoparticles. These particles were taken up by DCs in vitro. Immunization of C57BL/6 mice with these particles induced CD4^+^ as well as CD8^+^ T cells that produced IFNγ as well as the generation of antibodies. Moreover, ScaA/nanoparticle immunization protected the animals against lethal challenge with *O. tsutsugamushi* [158]. The use of nanoparticles for vaccine development has gained interest in the past years because nanoparticles can stabilize antigens and enhance the uptake of antigen by APCs, and in this way, overcome otherwise probably low immunogenicity. Furthermore, the use of nanoparticles allows targeted antigen delivery and slow release [159]. However, there are no further descriptions of the use of nanoparticles for experimental immunization against rickettsia.

### 6.3. Immunization with Nucleotides

One possibility of cytotoxic CD8^+^ and CD4^+^ T_H_ cell-oriented vaccination is the use of DNA. DNA immunization has been proven in various animal models of infections to efficiently induce cellular immunity. Upon intramuscular or intradermal application, the DNA is taken up by muscle cells and monocytes that then start to express the encoded protein. Intracellular cytosolic processing of the protein results in the generation of antigenic peptides that are presented by MHCI molecules for recognition by CD8^+^ T cells. In addition, CD4^+^ T cells can be induced by APCs that engulf the protein when released from the cells and present antigenic peptides via MHCII molecules [160,161].

DNA vaccination has been successfully applied for the induction of protective immunity against SFG rickettsiae and *O. tsutsugamushi* in experimental murine infection models. Heterologous prime-boost immunization was used for vaccination against SFG rickettsiae. DNA encoding for fragments of the OmpA protein (OmpA_703–1288_, OmpA_755–1301_ or OmpA_980–1301_ or OmpA_1644–2213_*)* from *R. conorii* in addition to a plasmid encoding for IL-12 was used for primary immunization followed by boost immunization with the corresponding recombinant protein fragments. In this way, lymphocytes were induced that produced IFNγ upon in vitro restimulation with *R. conorii* whole-cell antigen, and the mice were protected against normally lethal challenge with *R. conorii* [162,163]. Similarly, primary immunization with DNA encoding for fragments of the OmpB protein from *R. conorii* (OmpB_451–846_ or OmpB_754–1308_) followed by boost immunization with the corresponding recombinant protein fragments led to the same result, and combined DNA immunization with plasmids encoding for four protein fragments of OmpA and OmpB (OmpA_703–1288_ and OmpA_1644–2213_ or OmpB_451–846_ and OmpB_754–1308_) was protective against lethal challenge with the pathogen [163]. In the case of *O. tsutsugamushi*, DNA encoding for the Sta56 protein was used for the immunization of mice. Here, single immunization with the plasmid vector was not sufficient for protection, while partial protection (60% of the mice) was achieved after four immunizations with plasmid DNA [164].

The application of DNA, however, bears the risk of integration and persistence of the introduced DNA in the cellular genome. In addition, DNA vaccination can induce the generation of anti-DNA antibodies that can have serious side effects. A more elegant, safer, and modern way is the immunization with mRNA, which is shortly described in the following and reviewed in more detail elsewhere [165]. The mRNA encodes for an antigen or a part of an antigen and instructs the cells to transiently produce the encoded protein without the need to pass the nucleus membrane and without integration into the cellular genome. Conventional mRNAs carry the coding sequence of an antigen flanked by regulatory regions. Another form of mRNA vaccine is based on the modification of the genome of positive-stranded RNA viruses to obtain self-amplifying mRNA encoding for the antigen of choice, which ensures prolonged and robust expression of the antigen and subsequently better induction of adaptive immune responses. Conventional as well as self-amplifying mRNA vaccines are usually delivered packed with lipid nanoparticles (LNPs) as a vehicle that enhances the uptake of the material into the cell. Another method of delivery is the complexation of the mRNA with nucleotide-binding peptides such as protamine that stabilizes the mRNA and enhances its uptake into the cell. Apart from that, protamine acts as adjuvants by activating innate cells via the pattern recognition receptors (PRR) TLR7 and 8 [166,167,168]. In addition, bacterial and viral RNAs have been shown to be recognized by TLRs 3 and 7 [169], and in this way, possess an intrinsic adjuvant effect themselves [170,171]. This adjuvant effect, especially on professional APCs, is needed for efficient induction of adaptive immune response, and the complexation of mRNA with protamine has been demonstrated to enhance cytotoxic CD8^+^ T cell and CD4^+^ T_H_1 responses [167].

As these responses are also desired in protection against rickettsiae, mRNA vaccination is a promising strategy but has not been applied yet. Generally, the design of DNA as well as mRNA vaccines is quite flexible and offers the opportunity to combine several antigenic determinants from different proteins to obtain a broader spectrum of antigen-specific adaptive immune responses.

Regarding DNA and also mRNA immunization, one could also think of constructs that encode for a combination of antigenic determinants from different proteins, probably as a fusion protein. For example, CD4^+^ T cell epitopes have been identified from the *R. rickettsii* OmpB protein (OmpB_152–166_ (QNVVVQFNNGAAIDN), OmpB_399–413_ (NTDFGNLAAQIKVPN), OmpB_563–577_ (TIDLQANGGTIKLTS), OmpB_698–712_ (TNPLAEINFGSKGVN) and OmpB_1411–1425_ (NLMIGAAIGITKTDI)) and from the *R. rickettsii* YbgF protein (YbgF_57–71_ (LQHKIDLLTQNSNIS) [140]. These peptides, used alone or pooled or expressed as a recombinant fusion protein, induced protective immunity in C3H/HeN mice with the induction of CD4^+^ T_H_1 cells and antibody response [140]. A comparable vaccine that primarily addresses CD4^+^ T cells may also be sufficient for protection against *R. typhi*, and DNA or mRNA constructs could be designed for the expression of such fusion proteins.

Furthermore, five CD8^+^ T cell epitopes from the *R. conorii* OmpB protein have been described (OmpB_708–716_ (SKGVNVDTV), OmpB_789–797_ (ANSTLQIGG), OmpB_812–820_ (IVEFVNTGP), OmpB_735–743_ (ANVGSFVFN), and OmpB_749–757_ (IVSGTVGGQ) [172] that could also be integsrated into a fusion construct to obtain a CD8^+^ T cell response in addition to CD4^+^ T cells and antibody production.

The development of efficient DNA or mRNA vaccines may require codon-optimization to enable robust expression of rickettsial antigens in eukaryotic cells because the rickettsial genome possesses a very high A/T content. In addition, the efficacy of DNA and mRNA immunization can be generally significantly improved by several methods [173], e.g., the use of liposomes that facilitate the uptake into the cells after injection, the use of adjuvants or bicistronic constructs encoding for the antigen of choice in addition to costimulatory molecules or cytokines such as IL-12 that contribute to more efficient immune induction.

### 6.4. Vector-Based Immunization: Adenoviral Vectors

Genetically engineered replication-incompetent adenoviral vectors allow the efficient introduction of the transported genetic material into eukaryotic cells and have the potential to induce potent humoral as well as cellular responses. Different adenoviral vectors are in use for vaccination against SARS-Cov2, and adenoviral vectors have been studied as carriers for vaccinating antigens from several pathogens such as human immunodeficiency virus type I (HIV-1), *Plasmodium falciparum,* and *Mycobacterium* (*M*.) *tuberculosis* [174,175,176]. Different types of human adenoviral vectors have been studied. Human adenoviral vectors may be recognized by preexisting antibodies that are found in a very large proportion of the population. These antibodies can reduce the vector uptake and expression of the transgene, leading to reduced specific immune responses [177,178,179]. A chimpanzee adenoviral vector is an alternative. The immunizing effect of adenoviral vectors is generally very promising. Use for vaccination against rickettsiae has not been described yet, but should be taken into consideration in the future. This method offers similar opportunities as the design of DNA or mRNA vaccines with regard to flexibility in the combination of different antigens.

### 6.5. Vaccination with Genetically Modified Bacterial Vectors

*Mycobacterium* (*M*.) *vaccae* is an environmental member of the mycobacterial family and non-pathogenic for humans. It belongs to the same genus as *M. tuberculosis*, contains many homologous antigens, and is a promising vaccine in humans to prevent tuberculosis (e.g., Vaccae™ vaccine) used in an irradiation-killed or heat-killed form [180]. Immunization of mice with heat-killed *M. vaccae* itself induces cytotoxic CD8^+^ T cells that react to *M. tuberculosis*-infected MΦ and produce IFNγ and [181] and triggers a CD4^+^ T_H_1 response [182]. *M. vaccae* was also used for the expression of *M. tuberculosis* antigens. Applied to mice, such vaccine induces a T_H_1-biased *M. tuberculosis* antigen-specific response [183].

Similarly, genetically modified *M. vaccae* can potentially be used to induce immunity against other pathogens, including rickettsiae, as described in one study. Here, a plasmid encoding for fragments of the OmpA protein from *R. rickettsii* (OmpA_755–1301_ or OmpA_980–1301_) was introduced into *M. vaccae*. The engineered bacteria were then used for the immunization of C3H/HeN mice, followed by a boost immunization with recombinant OmpA_755–1301_ or OmpA_980–1301_ protein. In this way, IFNγ-producing rickettsia-specific T cells were induced, and the immunization mediated partial protection against challenge with *R. conorii* at a normally lethal dose [162].

### 6.6. Immunization with Antigen-Expressing Cells or Antigen-Pulsed APCs

Recently, CD8^+^ T cell antigens from R. prowazekii have been identified by bioinformatic approaches in reverse vaccinology (RP403, RP598, RP739, RP778, RP884) [131,132]. These antigens were recombinantly expressed in SVEC 4–10 cells to be presented by MHCI. Transfected SVEC 4–10 cells were then used for the immunization of C3H/HeN mice. The antigens were recognized by CD8^+^ T cells, and the immunization induced protective immunity to lethal challenge with *R. typhi* [131,132]. These are the only descriptions of the use of antigen-expressing cells for immunization, however.

Another possibility to induce CD4^+^ T cell responses is the use of professional APCs that are pulsed with recombinant antigenic proteins. This approach has been applied for immunization against the infection with *R. heilongjiangensis*. Bone marrow-derived DCs (bmDCs) from C3H/HeN mice were pulsed with recombinant fragments of the OmpB protein from the bacteria (OmpB_689–1033_, OmpB_991–1363_, OmpB_1346–1643_) and transferred into naïve C3H/HeN mice followed by challenge with *R. heilongjiangensis* 14 days afterward. The immunization resulted in reduced bacterial load and led to the activation of CD4^+^ as well as CD8^+^ T cells that produced IFNγ and TNFα, indicating a CD4^+^ T_H_1-biased and cytotoxic CD8^+^ T cell response [184].

These methods are highly interesting for the determination of immunogenic parts of a protein but would only be applicable for the immunization of individuals because the MHC haplotype must match for the recognition by T cells.

## 7. Conclusions

Several animal models for the infection with various rickettsiae have been used for experimental vaccination against these pathogens with different methods and some success. A limiting factor still is missing knowledge about immunogenic rickettsial determinants in general and especially about those that are recognized by T cells. As the cellular arm of the adaptive immune response clearly plays a dominant role in defense against rickettsial infections, such antigens need to be identified. Another focus should be the way of antigen delivery. So far, recombinant proteins and plasmid DNA immunization have been predominantly used for experimental vaccination of animals. Other promising ways of antigen delivery include the use of mRNA and adenoviral vectors, both of which are now successfully in use against the SARS-Cov2 pandemic. Other aspects that should be addressed and taken into consideration include the use of appropriate adjuvants and heterologous or homologous prime/boost regimens.

## Figures and Tables

**Table 1:** Family of *Rickettsiaceae*, rickettsial diseases and distribution. The table gives an overview of so-far-identified members of the family of Rickettsiaceae. SFG: spotted fever group, TG: typhus group, SF: spotted fever.

Genus	Group	Species	Disease	Distribution
***Rickettsia***	**SFG**	*R. rickettsii*	Rocky Mountain SF	North America (Midwest and Southeastern U.S.), Central and South America (Mexico, Panama, Costa Rica, Brazil, Argentina, Colombia)
		*R. conorii* ssp. *conorii*	Mediterranean SF	Europe (Mediterranean Basin), North Africa (Tunisia, Algeria, Morocco), multiple sub-Saharan countries
		*R. conorii* ssp. *indica*	Indian tick typhus	Middle East, India
		*R. conorii* ssp. *israelensis*	Israeli SF	Israel, North Africa (Tunisia)
		*R. conorii* ssp. *israelensis*	Astrakhan fever	Astrakhan region, France
		*R. conorii* ssp. *caspia*	Astrakhan fever	Africa (Chad)
		*364D (R. phillipi)*	Unnamed rickettsiosis	U.S. (southern California)
		*R. honei*	Flinder’s Island SF/Thailand SF	Australia, Tasmania, Thailand
		*R. helvetica*	Tick-bite fever	Europe (Denmark, Austria, France, Italy), Asia (Laos)
		*R. japonica*	Japanese SF	Japan, detected in ticks in South Korea and Northern Thailand
		*R. heilongjiangensis*	Far-Eastern SF	Northern China, Russia far east, Japan, Eastern Asia
		*R. parkeri*	Maculatum infection/American boutonneuse fever/Tidewater SF	North and South America
		*R. africae*	African tick-bite fever	Sub-Saharan Africa, Caribbean, West Indies
		*R. sibirica*	Siberian tick typhus/North Asian tick typhus	Russia, China, Mongolia
		*R. sibiria* ssp. *mongolotimonae*	Tickborne lymphadenopathy (TIBOLA)	Southern Europe (France, Greece, Portugal, Spain), Asia, South Africa
		*R. massiliae*	Mediterranean SF-like disease	Southern Europe, South America (Argentina)
		*R. monacensis*	Tick-bite fever	Europe
		*R. slovaca*	Tickborne lymphadenopathy (TIBOLA)/Dermacentor-borne necrosis and lymphadenopathy (DEBONEL)/ scalp eschar and neck lymphadenopathy after tick bite (SENLAT)	Europe (France, Slovakia, Italy, Germany, Hungary, Spain, Poland), Georgia, Russia
		*R. raoultii*		Europe (France, Slovakia, Poland)
		*R. aeschlimannii*	Tick-bite fever	North Africa (Tunisia, Morocco), South Africa
		*364D* (*R. phillipi*)	Unnamed rickettsiosis	Southern California
	**TG**	*R. prowazekii*	Epidemic typhus	Worldwide, sporadic in Africa, Asia, Central and South America, Russia
		*R. typhi*	Endemic typhus/Murine typhus	Worldwide
	**transitional**	*R. felis*	Cat-flea typhus	Probably worldwide
		*R. akari*	Rickettsialpox	Probably worldwide
		*R. australis*	Queensland tick typhus	Australia, Tasmania
	**ancestral**	*R. bellii*		
		*R. canadensis*		
***Orientia***		*O. tsutsugamushi*	Scrub typhus/tsutsugamushi fever	Asia, Northern Australia, serological evidence in sub-Saharan countries (Cameroon, Congo, Kenya)
		*candidatus O. chuto*	Scrub typhus/tsutsugamushi fever	Arabian Peninsula (Dubai), other areas in Middle East?
		*candidatus O. chiloensis*	Scrub typhus/tsutsugamushi fever	Chile

**Table 2:** Overview on experimentally identified rickettsial immunogens, their localization, and recognition by B and/or T cells. The table provides an overview of so-far-known antigens from rickettsiae and orientia, their localization and function, and whether they are recognized by B and/or T cells. OM: outer membrane, IM: inner membrane, C: cytoplasm, P: periplasm, EC: extracellular, **√**: experimentally proven recognition. Empty fields: not described.

Rickettsial	Localization	Function	Recognition by		
**Immunogens**			**B**	**CD4+**	**CD8+**
Sca0 (OmpA)	OM	adhesion and invasion	**√**	**√**	
Sca1	OM	adhesion and invasion	**√**		
Sca2	OM	adhesion and invasion	**√**		
Sca3	OM	adhesion and invasion	**√**		
Sca4	C	binds and activates vinculin [107]	**√**		
Sca5 (OmpB)	OM	adhesion and invasion	**√**	**√**	**√**
Adr1	OM	adhesion and invasion, binds vitronectin, confers resistance to complement-mediated killing [108,109]	**√**		
Adr2	OM	adhesion and invasion, binds vitronectin, confers resistance to complement-mediated killing [110]	**√**	**√**	**√**
TolC	OM	adhesion and invasion of vascular endothelial cells [111]	**√**		
OmpW	OM	adhesion and invasion of vascular endothelial cells [111]			
Porin-4	IM/OM/EC	export of glycostructures (eg. LPS O-antigen)	**√**		
YbgF	OM/C	tol-pal system protein	**√**	**√**	**√**
GroEL	C/OM }	60 kDa heat shock protein, molecular chaperone; surface-exposed [105,106,112]	**√**		
PrsA	OM/C	Parvulin-like peptidyl-prolyl cis-trans isomerase (Parvulin-like PPIase), protein export protein	**√**		
RplY	C/OM	50S ribosomal protein L25/general stress protein Ctc	**√**		
RpsB	C/OM	30S ribosomal protein S2	**√**		
SurA	C/OM	chaperone SurA, parvulin-like peptidyl-prolyl isomerase	**√**		
RP403	C/OM	RecB family exonuclease			**√**
RP598	C/OM	transcription repair coupling factor			**√**
RP739	IM	ADP/ATP carrier protein (tlc5)			**√**
RP778	C/OM	DNA polymerase III a chain (dnaE)			**√**
RP884	C	ferrochelatase (hemE)			**√**
**Orientia immunogens**	**Localization**	**Function**	**B**	**CD4^+^**	**CD8^+^**
Sta22	OM	TSA47, transposase/DegP-like serin protease	**√**	**√**	
Sta47	C/P	TSA56, multi-pass membrane protein	**√**		
Sta56	OM	autotransporter protein	**√**	**√**	
ScaA	OM	autotransporter protein	**√**	**√**	**√**
ScaC	OM	autotransporter protein	**√**		
ScaD	OM	autotransporter protein	**√**		
ScaE	C/OM	TSA47, transposase/DegP-like serin protease	**√**		

## Data Availability

The origin of all data mentioned in the text is cited in the reference list.
